# Bis(imidazo[1,2-*a*]pyridin-1-ium) tetra­chlorido­cuprate(II) dihydrate

**DOI:** 10.1107/S2056989017000482

**Published:** 2017-01-13

**Authors:** Sonia Mokaddem, Habib Boughzala

**Affiliations:** a Laboratoire de Matériaux et Cristallochimie, Faculté des Sciences de Tunis, Université de Tunis El Manar, 2092 Manar II Tunis, Tunisia

**Keywords:** crystal structure, copper(II), organic–inorganic hybrid

## Abstract

In the title salt, (C_7_H_7_N_2_)_2_[CuCl_4_]·2H_2_O, the Cu^2+^ cation is coordinated by four Cl atoms and adopts a distorted tetra­hedral geometry. Two mol­ecules of imidazo[1,2-*a*]pyridine are protonated ensuring electrical neutrality. O—H⋯Cl and N—H⋯O hydrogen bonds link the organic and the inorganic moieties, leading to a self-organized hydrated hybrid structure.

## Abstract 2

Copper is known for its variable coordination geometry adopted for different ligands. Here we report the crystal structure of a new imidazo[1,2-*a*]pyridin-1-ium tetra­chlorido­cuprate(II) salt with a distorted tetra­hedral geometry for the copper atom.

## Chemical context   

Copper halides have applications in biology as anti­fungal and anti­cancer agents (Creaven *et al.*, 2010 ▸; Santini *et al.*, 2014 ▸) and are also good precursors for photovoltaic cells because of their optoelectronic and magnetic properties (Levitsky *et al.*, 2004 ▸; Ahmadi *et al.*, 2013 ▸; Al-Far & Ali, 2009 ▸). For this reason, we have focused our research on copper-based hybrid materials using diverse organic moieties to balance the halide copper inorganic anions. We report in this paper the synthesis and structure determination using single crystal X-ray diffraction data of a tetrahedral tetrachloridocuprate(II) anion with imidazo[1,2-*a*]pyridin-1-ium organic cations and two lattice water molecules.
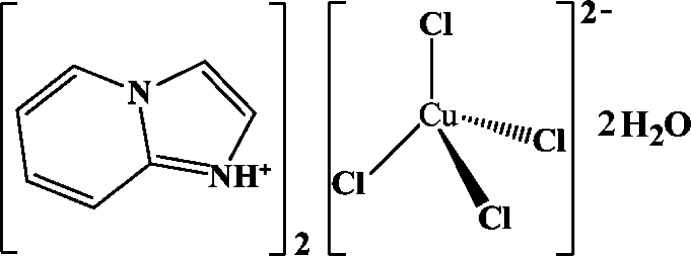


## Structural commentary   

**Figure 1:**
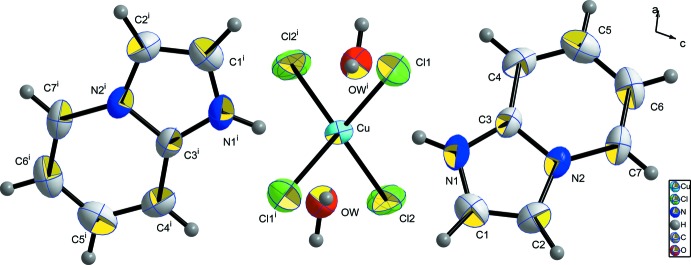
*ORTEP*-style plot of the structural unit with displacement ellipsoids at the 50% probability level. [Symmetry code: (i) 1 − *x*, *y*, 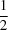 − *z*.]

The structural unit (Fig. 1 ▸) of the title compound comprises one [CuCl_4_]^2−^ anion, two organic imidazo[1,2-*a*]pyridine ligands and two water mol­ecules.

When coordinated by halide anions, copper can adopt several coordination geometries including tetra­hedral, square-pyramidal, square-planar and square-bipyramidal (Bhattacharya *et al.*, 2004 ▸; Yuan *et al.*, 2004 ▸). A four-coordinate geometry is generally inter­mediate between square-planar and regular-tetra­hedral, as reported by Al-Far & Ali (2009 ▸). In our case and according to the angular values of the copper–chlorine bonds, summarized in Table 1 ▸, the tetra­hedral copper coordination seems to be slightly distorted. These distortions are a consequence of the lower mol­ecular symmetry.

The (C_7_H_7_N_2_)^+^ cation adopts a quite planar conformation, as characterized by its low r.m.s deviation of 0.0064 Å. The maximum deviations are 0.010 (3) and −0.012 (2) Å for atoms C1 and N2, respectively.

**Figure 2:**
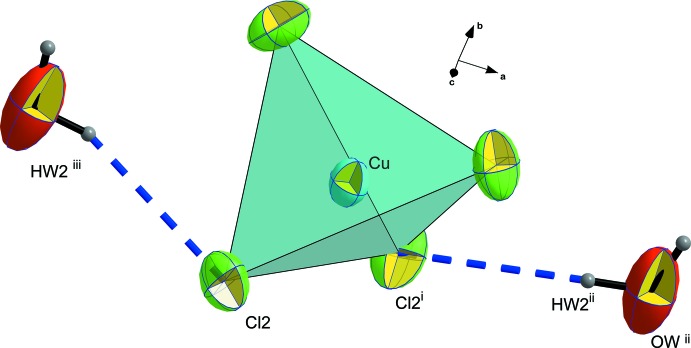
The [CuCl_4_]^2−^ environment with hydrogen bonds shown as blue dashed lines. Displacement ellipsoids are displayed at the 50% probability level. [Symmetry codes: (i) 1 − *x*, *y*, 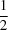 − *z*; (ii) 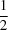 + *x*, 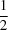 + *y*, *z*; (iii) 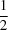 − *x*, 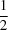 + *y*, 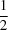 − *z*.]

The two water mol­ecules are located approximately in a common plane defined by the organic cations, directing their hydrogen atoms towards the anionic group [CuCl_4_]^2−^ and leaving the oxygen free-electron pairs available for a hydrogen-bonding inter­action with the protonated nitro­gen site of the imidazo[1,2-*a*]pyridinum cation. In the anionic subnetwork, every [CuCl_4_]^2−^ anion is linked to two water mol­ecules by hydrogen bonds *via* the Cl2 vertices, as shown in Fig. 2 ▸.

**Figure 3:**
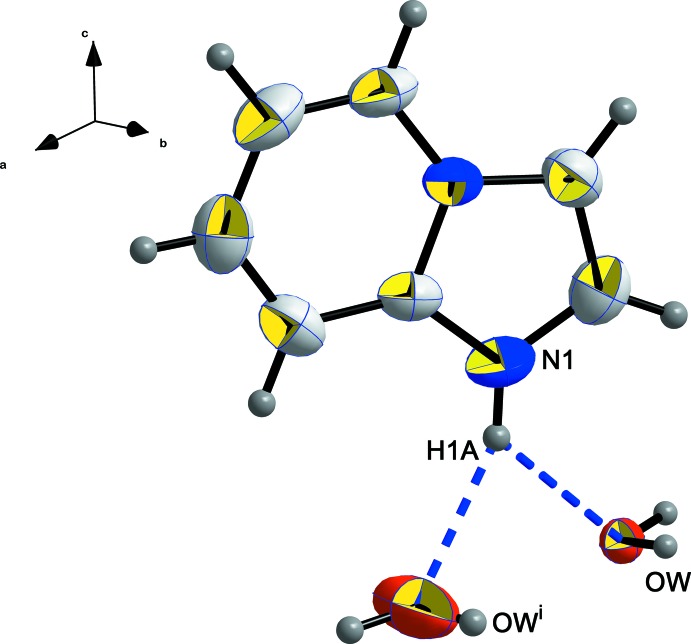
The environment around the (C_7_H_7_N_2_)^+^ cation showing the inter­actions with water mol­ecules through N1—H1*A*⋯O*W* and N1—H1*A*⋯O*W* ^i^ inter­actions. Displacement ellipsoids are displayed at the 50% probability level. [Symmetry code: (i) 1 − *x*, *y*, 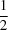 − *z*.]

**Figure 4:**
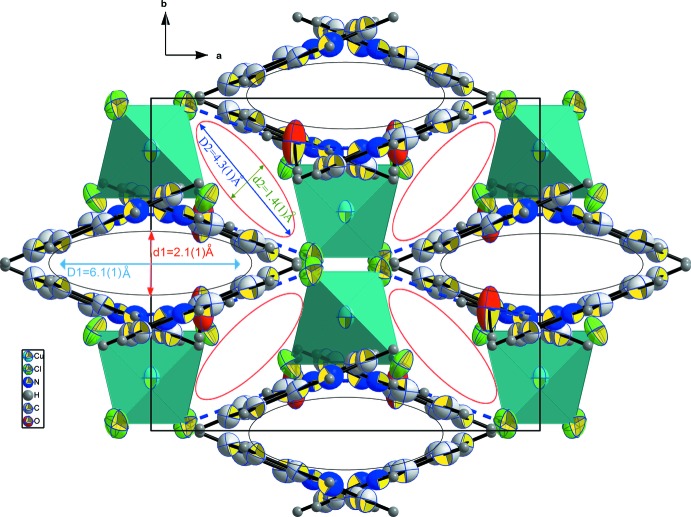
Crystal packing along the *c* axis showing empty tunnels able to lodge small organic solvent mol­ecules. Displacement ellipsoids are displayed at the 50% probability level.

In spite of the single protonation of the organic mol­ecule on the aromatic nitro­gen site, every cation is linked to two water mol­ecules through bifurcated hydrogen-bonding inter­actions along [010], as shown in Fig. 3 ▸. The organic cations are organized along (010), forming sheets parallel to the *ab* plane. A projection of the structural packing along the *c* axis, Fig. 4 ▸, reveals alternating empty elliptical channels delimited by the organic cations and inorganic tetra­hedra. The long and short dimensions of the elliptical sections are estimated to be, respectively, 6.1 (1) and 2.1 (1) Å for the largest ones and 4.3 (1) and 1.4 (1) Å for the narrowest. These voids are able to lodge several small solvent mol­ecules.

**Figure 5:**
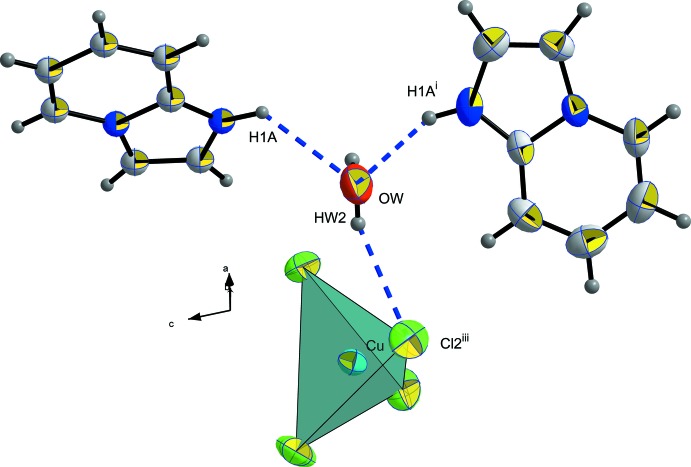
The environment around the water mol­ecule. Hydrogen bonds are indicated by blue dashed lines. Displacement ellipsoids are displayed at the 50% probability level. [Symmetry codes: (i) 1 − *x*, *y*, 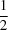 − *z*; (iii) 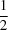 − *x*, −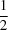 + *y*, 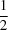 − *z*.]

**Figure 6:**
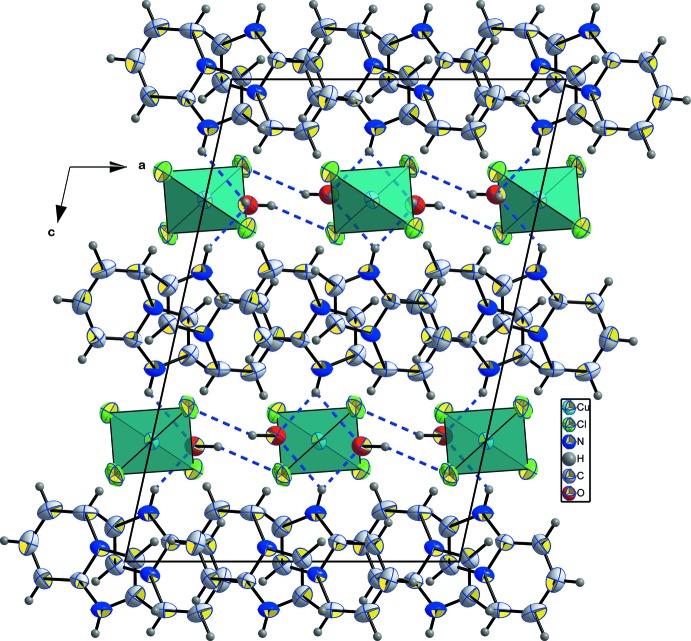
View of the packing showing the alternating stacking of the organic and inorganic layers connected through hydrogen bonds. The face-to-face π–π stacking between parallel organic mol­ecules is noteworthy with a centroid–centroid distance of 3.968 (3) Å. Displacement ellipsoids are displayed at the 50% probability level. [Symmetry code: (iii) 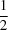 − *x*, −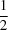 + *y*, 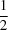 − *z*.]

The water mol­ecules play a crucial role in the crystal-packing cohesion. Every water mol­ecule is linked to one [CuCl_4_] ^2−^ tetra­hedron through O—H⋯Cl hydrogen bonds (Table 2 ▸) and to two organic mol­ecules through O—H⋯N hydrogen bonds, as shown in Fig. 5 ▸. The expected structural self-organization generally present in hybrid inorganic–organic compounds can also be found in the structure of the title salt. The alternating stacking of organic and inorganic sheets observed along the *c* axis (Fig. 6 ▸) could possibly lead to luminescence properties.

## Supra­molecular features   

The lowering of the symmetry of the copper coordination could also be due to halide–halide and intra- and intermol­ecular hydrogen-bonding inter­actions; these inter­actions are closely related to the shape and the size of the counter-cations (Bouacida *et al.*, 2013 ▸; Parent *et al.*, 2007 ▸; Haddad *et al.*, 2006 ▸; Marzotto *et al.*, 2001 ▸; Choi *et al.*, 2002 ▸; Awwadi *et al.*, 2007 ▸). Non-covalent inter­actions such as hydrogen-bonding inter­actions and π–π stacking inter­actions represent the most important linkers in this kind of material. Moreover, these inter­actions are able to delimit not only the architecture, but also impact on the properties of metal–halide materials. The organic cations are linked to the water mol­ecule through N1—H1*A*⋯O*W* hydrogen bonds (Table 2 ▸) and are connected through face-to-face π–π stacking [*Cg*1⋯*Cg*2(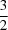 − *x*, 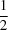 − *y*, 1 − *z*) = 3.968 (3) Å where *Cg*1 and *Cg*2 are the centroids of the N1/N3/C1–C3 and N2/C3–C7 rings, respectively]. The crystal packing can be described by alternating stacks of anions and cationic chains with the organic layers arranged parallel to the anionic stacks.

## Database survey   

Imidazo[1,2-*a*]-pyridyn-1-ium cations and several substituted forms have 53 entries in the Cambridge Structural Database (Groom *et al.*, 2016 ▸) without any hybrid compounds amongst them. To the best of our knowledge, this work is the first chemical and crystallographic identification of tetrachloridocuprate(II) combined with imidazo[1,2-*a*]-pyridyn-1-ium.

## Synthesis and crystallization   

The title salt was prepared by the reaction of imidazo[1,2-*a*]pyridine and Cu(NO_3_)_2_·2H_2_O (molar ratio 1:1) in an equal volume of water and ethanol (10 ml) mixed with 2 ml of hydro­chloric acid (37%). The solution was stirred for 1 h at 333 K. Prismatic yellow crystals suitable for X-ray diffraction were grown in one week by slow evaporation at room temperature.

## Refinement   

Crystal data, data collection and structure refinement details are summarized in Table 3 ▸. H atoms were positioned geometrically and treated as riding on the parent atom with C—H = 0.93 Å and N—H = 0.86 Å. For H*W*1 and H*W*2, the restraints DFIX and DANG were used to stabilize the water mol­ecule.

## Supplementary Material

Crystal structure: contains datablock(s) I. DOI: 10.1107/S2056989017000482/vn2122sup1.cif

Structure factors: contains datablock(s) I. DOI: 10.1107/S2056989017000482/vn2122Isup2.hkl

CCDC reference: 1526513

Additional supporting information:  crystallographic information; 3D view; checkCIF report

## Figures and Tables

**Table 1:** Selected geometric parameters (Å, °)

Cu—Cl2	2.2100 (11)	Cu—Cl1	2.2499 (13)
			
Cl2—Cu—Cl2^i^	105.26 (7)	Cl2—Cu—Cl1	121.58 (4)
Cl2—Cu—Cl1^i^	102.86 (5)	Cl1^i^—Cu—Cl1	104.12 (7)

Symmetry code: (i) 
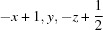
.

**Table 2:** Hydrogen-bond geometry (Å, °)

*D*—H⋯*A*	*D*—H	H⋯*A*	*D*⋯*A*	*D*—H⋯*A*
N1—H1*A*⋯O*W*	0.86	2.50	3.0723	125
N1—H1*A*⋯O*W* ^i^	0.86	2.08	2.872	152 (1)
O*W*—H*W*2⋯Cl2^ii^	0.85	2.61 (1)	3.401	157 (1)

Symmetry codes: (i) 
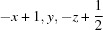
; (ii) 
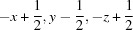
.

**Table 3:** Experimental details

Crystal data	
Chemical formula	(C_7_H_7_N_2_)_2_[CuCl_4_]·2H_2_O
*M*_r_	479.66
Crystal system, space group	Monoclinic, *C*2/*c*
Temperature (K)	298
*a*, *b*, *c* (Å)	11.747 (8), 9.793 (2), 17.339 (4)
β (°)	102.48 (5)
*V* (Å^3^)	1947.7 (15)
*Z*	4
Radiation type	Mo *K*α
μ (mm^−1^)	1.69
Crystal size (mm)	0.45 × 0.15 × 0.1

Data collection	
Diffractometer	Enraf–Nonius CAD-4
Absorption correction	ψ scan (North *et al.*, 1968 ▸).
*T*_min_, *T*_max_	0.746, 0.845
No. of measured, independent and observed [*I* > 2σ(*I*)] reflections	3358, 2127, 1796
*R*_int_	0.042
(sin θ/λ)_max_ (Å^−1^)	0.638

Refinement	
*R*[*F* ^2^ > 2σ(*F* ^2^)], *wR*(*F* ^2^), *S*	0.038, 0.108, 1.04
No. of reflections	2127
No. of parameters	120
No. of restraints	3
H-atom treatment	H atoms treated by a mixture of independent and constrained refinement
Δρ_max_, Δρ_min_ (e Å^−3^)	0.78, −0.49

Computer programs: *CAD-4 EXPRESS* (Enraf–Nonius, 1994 ▸), *XCAD4* (Harms & Wocadlo, 1995 ▸), *SHELXS97* (Sheldrick, 2008 ▸), *SHELXL2014* (Sheldrick, 2015 ▸), *DIAMOND* (Brandenburg, 2006 ▸) and *publCIF* (Westrip, 2010 ▸).

## supplementary crystallographic information

### Crystal data

**Table:**

(C_7_H_7_N_2_)_2_[CuCl_4_]·2H_2_O	*F*(000) = 972
*M**_r_* = 479.66	*D*_x_ = 1.636 Mg m^−^^3^
Monoclinic, *C*2/*c*	Mo *K*α radiation, λ = 0.71073 Å
*a* = 11.747 (8) Å	Cell parameters from 25 reflections
*b* = 9.793 (2) Å	θ = 2–27°
*c* = 17.339 (4) Å	µ = 1.69 mm^−^^1^
β = 102.48 (5)°	*T* = 298 K
*V* = 1947.7 (15) Å^3^	Prism, yellow
*Z* = 4	0.45 × 0.15 × 0.1 mm

### Data collection

**Table:**

Enraf–Nonius CAD-4 diffractometer	*R*_int_ = 0.042
Radiation source: fine-focus sealed tube	θ_max_ = 27.0°, θ_min_ = 2.4°
non–profiled ω/2τ scans	*h* = −14→4
Absorption correction: ψ scan (North *et al.*, 1968).	*k* = −1→12
*T*_min_ = 0.746, *T*_max_ = 0.845	*l* = −22→22
3358 measured reflections	2 standard reflections every 120 min
2127 independent reflections	intensity decay: 32%
1796 reflections with *I* > 2σ(*I*)	

### Refinement

**Table:**

Refinement on *F*^2^	3 restraints
Least-squares matrix: full	Hydrogen site location: mixed
*R*[*F*^2^ > 2σ(*F*^2^)] = 0.038	H atoms treated by a mixture of independent and constrained refinement
*wR*(*F*^2^) = 0.108	*w* = 1/[σ^2^(*F*_o_^2^) + (0.0535*P*)^2^ + 3.2027*P*] where *P* = (*F*_o_^2^ + 2*F*_c_^2^)/3
*S* = 1.04	(Δ/σ)_max_ = 0.001
2127 reflections	Δρ_max_ = 0.78 e Å^−^^3^
120 parameters	Δρ_min_ = −0.49 e Å^−^^3^

### Special details

**Table:**

Geometry. All esds (except the esd in the dihedral angle between two l.s. planes) are estimated using the full covariance matrix. The cell esds are taken into account individually in the estimation of esds in distances, angles and torsion angles; correlations between esds in cell parameters are only used when they are defined by crystal symmetry. An approximate (isotropic) treatment of cell esds is used for estimating esds involving l.s. planes.
Refinement. Refinement of F^2^ against ALL reflections. The weighted R-factor wR and goodness of fit S are based on F^2^, conventional R-factors R are based on F, with F set to zero for negative F^2^. The threshold expression of F^2^ > 2sigma(F^2^) is used only for calculating R-factors(gt) etc. and is not relevant to the choice of reflections for refinement. R-factors based on F^2^ are statistically about twice as large as those based on F, and R- factors based on ALL data will be even larger.

### Fractional atomic coordinates and isotropic or equivalent isotropic displacement parameters (Å^2^)

**Table:**

	*x*	*y*	*z*	*U*_iso_*/*U*_eq_	
Cu	0.5000	0.65843 (5)	0.2500	0.03734 (16)	
Cl1	0.64409 (8)	0.79968 (10)	0.30835 (5)	0.0654 (3)	
Cl2	0.41162 (7)	0.52147 (11)	0.31978 (6)	0.0691 (3)	
N1	0.5383 (2)	0.1611 (3)	0.39993 (14)	0.0503 (6)	
H1A	0.5411	0.1555	0.3509	0.060*	
N2	0.5837 (2)	0.1477 (2)	0.52756 (13)	0.0407 (5)	
C1	0.4463 (3)	0.2105 (4)	0.4280 (2)	0.0564 (8)	
H1	0.3770	0.2439	0.3973	0.068*	
C2	0.4724 (3)	0.2029 (3)	0.50690 (19)	0.0508 (7)	
H2	0.4254	0.2294	0.5411	0.061*	
C3	0.6232 (2)	0.1228 (3)	0.46046 (15)	0.0411 (6)	
C4	0.7339 (3)	0.0682 (3)	0.4645 (2)	0.0522 (7)	
H4	0.7615	0.0503	0.4191	0.063*	
C5	0.8004 (3)	0.0420 (4)	0.5383 (2)	0.0608 (8)	
H5	0.8747	0.0056	0.5433	0.073*	
C6	0.7581 (3)	0.0691 (4)	0.6061 (2)	0.0614 (9)	
H6	0.8047	0.0507	0.6556	0.074*	
C7	0.6512 (3)	0.1214 (3)	0.60080 (17)	0.0528 (7)	
H7	0.6234	0.1394	0.6461	0.063*	
OW	0.3680 (2)	0.1331 (5)	0.23911 (17)	0.0959 (12)	
HW1	0.376 (5)	0.2194 (15)	0.244 (5)	0.144*	
HW2	0.2977 (19)	0.114 (5)	0.238 (4)	0.144*	

### Atomic displacement parameters (Å^2^)

**Table:**

	*U*^11^	*U*^22^	*U*^33^	*U*^12^	*U*^13^	*U*^23^
Cu	0.0303 (2)	0.0469 (3)	0.0372 (2)	0.000	0.01252 (17)	0.000
Cl1	0.0549 (5)	0.0787 (6)	0.0612 (5)	−0.0256 (4)	0.0092 (4)	−0.0132 (4)
Cl2	0.0553 (5)	0.0861 (6)	0.0731 (5)	−0.0074 (4)	0.0300 (4)	0.0268 (5)
N1	0.0616 (15)	0.0559 (15)	0.0321 (11)	−0.0059 (12)	0.0070 (11)	0.0009 (10)
N2	0.0518 (13)	0.0387 (12)	0.0339 (10)	−0.0029 (10)	0.0143 (10)	0.0000 (9)
C1	0.0509 (17)	0.0544 (18)	0.0594 (19)	−0.0002 (14)	0.0022 (14)	0.0033 (15)
C2	0.0522 (17)	0.0501 (16)	0.0544 (17)	0.0015 (13)	0.0208 (14)	0.0002 (14)
C3	0.0502 (15)	0.0410 (13)	0.0337 (12)	−0.0089 (11)	0.0128 (11)	−0.0028 (11)
C4	0.0546 (17)	0.0505 (16)	0.0570 (17)	−0.0064 (13)	0.0239 (14)	−0.0077 (14)
C5	0.0485 (17)	0.0515 (18)	0.079 (2)	−0.0010 (14)	0.0060 (16)	−0.0004 (17)
C6	0.074 (2)	0.0547 (18)	0.0467 (17)	−0.0044 (16)	−0.0062 (16)	0.0051 (15)
C7	0.074 (2)	0.0523 (17)	0.0312 (13)	−0.0027 (15)	0.0085 (13)	0.0015 (12)
OW	0.0537 (15)	0.186 (4)	0.0483 (14)	−0.0107 (19)	0.0127 (12)	0.010 (2)

### Geometric parameters (Å, º)

**Table:**

Cu—Cl2	2.2100 (11)	C2—H2	0.9300
Cu—Cl2^i^	2.2100 (11)	C3—C4	1.394 (4)
Cu—Cl1^i^	2.2499 (13)	C4—C5	1.372 (5)
Cu—Cl1	2.2499 (13)	C4—H4	0.9300
N1—C3	1.336 (4)	C5—C6	1.397 (6)
N1—C1	1.366 (5)	C5—H5	0.9300
N1—H1A	0.8600	C6—C7	1.340 (5)
N2—C3	1.364 (3)	C6—H6	0.9300
N2—C7	1.369 (4)	C7—H7	0.9300
N2—C2	1.388 (4)	OW—HW1	0.854 (10)
C1—C2	1.338 (5)	OW—HW2	0.845 (10)
C1—H1	0.9300		
			
Cl2—Cu—Cl2^i^	105.26 (7)	N2—C2—H2	126.8
Cl2—Cu—Cl1^i^	102.86 (5)	N1—C3—N2	106.6 (3)
Cl2^i^—Cu—Cl1^i^	121.58 (4)	N1—C3—C4	132.6 (3)
Cl2—Cu—Cl1	121.58 (4)	N2—C3—C4	120.7 (3)
Cl2^i^—Cu—Cl1	102.86 (5)	C5—C4—C3	117.1 (3)
Cl1^i^—Cu—Cl1	104.12 (7)	C5—C4—H4	121.5
C3—N1—C1	109.5 (3)	C3—C4—H4	121.5
C3—N1—H1A	125.3	C4—C5—C6	121.0 (3)
C1—N1—H1A	125.3	C4—C5—H5	119.5
C3—N2—C7	121.5 (3)	C6—C5—H5	119.5
C3—N2—C2	108.9 (2)	C7—C6—C5	120.8 (3)
C7—N2—C2	129.6 (3)	C7—C6—H6	119.6
C2—C1—N1	108.7 (3)	C5—C6—H6	119.6
C2—C1—H1	125.7	C6—C7—N2	118.9 (3)
N1—C1—H1	125.7	C6—C7—H7	120.6
C1—C2—N2	106.3 (3)	N2—C7—H7	120.6
C1—C2—H2	126.8	HW1—OW—HW2	108 (3)

Symmetry code: (i) −*x*+1, *y*, −*z*+1/2.

### Hydrogen-bond geometry (Å, º)

**Table:**

*D*—H···*A*	*D*—H	H···*A*	*D*···*A*	*D*—H···*A*
N1—H1*A*···O*W*	0.86	2.50	3.0723	125
N1—H1*A*···O*W*^i^	0.86	2.08	2.872	152 (1)
O*W*—H*W*2···Cl2^ii^	0.85	2.61 (1)	3.401	157 (1)

Symmetry codes: (i) −*x*+1, *y*, −*z*+1/2; (ii) −*x*+1/2, *y*−1/2, −*z*+1/2.
